# Automatic mode switching in atrial fibrillation

**Published:** 2005-07-01

**Authors:** Giuseppe Stabile, Antonio De Simone, Enrico Romano

**Affiliations:** Laboratorio di Elettrofisiologia, Casa di Cura San Michele, Maddaloni (CE), Italia; * St Jude Medical Italia

**Keywords:** automatic mode switching, atrial fibrillation

## Abstract

Automatic mode switching (AMS) algorithms were designed to prevent tracking of atrial tachyarrhythmias (ATA) or other rapidly occurring signals sensed by atrial channels, thereby reducing the adverse hemodynamic and symptomatic consequences of a rapid ventricular response. The inclusion of an AMS function in most dual chamber pacemaker now provides optimal management of atrial arrhythmias and allows the benefit of atrioventricular synchrony to be extended to a population with existing atrial fibrillation. Appropriate AMS depends on several parameters: a) the programmed parameters; b) the characteristics of the arrhythmia; c) the characteristics of the AMS algorithm. Three qualifying aspects constitute an AMS algorithm: onset, AMS response, and resynchronization. Since AMS programs also provide data on the time of onset and duration of AMS episodes, AMS data may be interpreted as a surrogate marker of ATAs recurrence. Recently, stored electrograms corresponding to episodes of ATAs have been introduced, thus clarifying the accuracy of AMS in detecting ATAs Clinically this information may be used to assess the efficacy of an antiarrhythmic intervention or the risk of thromboembolic events, and it may serve as a valuable research tool  for evaluating the natural history and burden of ATAs.

In early generation of dual-chamber pacemakers (PMs) paroxysmal atrial tachyarrhythmias (ATAs) were considered a contraindication to pacing modes with atrial tracking due to concerns of rapid ventricular tracking of atrial arrhythmias. Automatic mode switching (AMS) was first introduced as a trademark in implantable devices in 1993. AMS algorithms were designed to prevent tracking of ATAs or other rapidly occurring signals sensed by atrial channels, thereby reducing the adverse hemodynamic and symptomatic consequences of a rapid ventricular response [1] (Figure 1). The inclusion of an AMS function in most dual chamber PMs now provides optimal management of atrial arrhythmias and allows the benefit of atrioventricular synchrony to be extended to a population with existing atrial fibrillation (AF). The devices of the present generation, indicated in all patients with the brady-tachy syndrome, should also be considered in patients with sinus node disease without paroxysmal AF, obstructive hypertrophic cardiomyopathy, or any condition that predisposes patients to paroxysmal AF [2]. Clinical studies have shown that the incidence of ATAs and AMS in patients with dual chamber PMs is high [3,4]. Since AMS programs also provide data on the time of onset and duration of AMS episodes, AMS data may be interpreted a surrogate marker of ATAs recurrence. Clinically this information may be used to assess the efficacy of an antiarrhythmic intervention or the risk of thromboembolic events, and it may serve as a valuable research tool  for evaluating the natural history and burden of ATAs. This article analyzes AMS concepts with regard to their algorithms, clinical impact and useful for ATA diagnosis in patients with permanent PMs.

## Automatic mode switching algorithms

An optimal AMS algorithm should provide a high sensitivity and specificity for the detection of atrial arrhythmias, maintain atrioventricular synchrony, prevent the triggering of atrial arrhythmias and facilitate rapid resumption of atrioventricular synchrony when the arrhythmia ceases. When it is instrumented on a high voltage antiarrhythmic device, two more functions are required: the withholding of inappropriate ventricular therapies and the delivery of atrial therapies.

Many algorithms have been used by different manufacturers, in recent years, and, of course, the clinical behaviour differs according to the pacemaker models.

Appropriate AMS depends on several parameters (Table): a) the programmed parameters; b) the characteristics of the arrhythmia; c) the characteristics of the AMS algorithm.

In order to avoid inappropriate AMS a customized optimal care of the patient would be required, with a thorough knowledge of the arrhythmia history, of the atrial signal amplitude (mostly during atrial arrhythmia) and of the required programming parameters (i.e. the atrial post-ventricular blanking period) and the characteristics of the available AMS algorithms. Indeed AMS failure may occur if the amplitude of the atrial electrogram is intermittently or consistently too small to be sensed or if an atrial signal occurs systematically during the atrial blanking period [6]. Appropriate programming of atrial sensitivity, and the avoidance of ventriculo-atrial cross-talk, near and far field and other fake signals are essential for optimal AMS performance.

Three qualifying aspects constitute an AMS algorithm [7]:

*Onset:* it is worthwhile to characterize the speed of response of the algorithm at arrhythmia onset. It can be expressed in time or number of cycles. Rapid response algorithms exhibit rate instability and slow response algorithms exhibit long delay in response, hence risk of atrioventricular dissociation. A fast AMS might be capable of preventing symptoms not only in patients with underlying atrioventricular block, but also in those with intact atrioventricular conduction, as it avoids high ventricular paced rates at the onset of the arrhythmia.

*AMS response:* this is the ability of the algorithm to obtain ventricular pacing rate regularization during the period between the onset of AMS and the termination of atrial arrhythmia. In several PMs models, after the mode switch event, a different base rate could be programmed in the ventricle. This provides a programmable elevated pacing rate to compensate for the loss of atrial transport during periods of ATA, while the device is in a non-tracking mode. Furthermore, it is quite always possible to program a cross-sensor feature, switching from a non-rate-responsive mode to a sensor mode or vice versa.

*Resynchronization:* it is defined as the response to normal sinus rhythm resumption or arrhythmia ending, thus the time latency or the number of beats needed to recover the atrioventricular tracking functioning.

It is clear that speed of response and rate stability are two independent and, unfortunately, competing parameters. Also the termination response is independent from the onset response, in fact shorter reaction times of the algorithm might be used without an increased risk for inappropriate termination of mode switch. Some AMS algorithms use the same onset criteria to resynchronize after arrhythmia termination, whereas others use slower criteria of resynchronization to avoid intermittent AMS during short runs of arrhythmia.

The type of nontracking mode and the adequacy of ventricular rate during AMS determine the clinical efficacy of AMS [7]. Either VVI or DDI mode is used. The VVI mode during AMS has been described as VDI because the maintenance of atrial sensing allows the perpetuation or termination of AMS. Obviously during AMS there is no AV synchrony and the DDI mode is functionally equivalent to the VVI mode. The DDI mode offers the advantage of atrial pacing as soon as ATA terminates even if criteria for detection of ATA termination by the device have not yet been met, thus avoiding AV dissociation when a sinus pause occurs at ATA termination. This may result in PM mediated tachycardia if retrograde conduction is present, may provoke PM syndrome, and occasionally may reinduce ATA. On the other hand when AF is undersensed during AMS, atrial pacing in the DDI destination mode may paradoxically perpetuate AF [8]. A sudden rate drop at the start of AMS can cause symptoms and decrease cardiac output [7]. Therefore, rate smoothing algorithms gradually decrease the ventricular rate from the rate at the moment of AMS to the sensor rate or the lower rate. In patients with complete AV block, AMS should be performed to a rate responsive mode, since otherwise pacing will be performed at the lower rate limit for long periods. This may not be adequate during physical activity [9].

Accuracy of AMS algorithms. The accuracy of an algorithm of detecting an ATA may be expressed in terms of sensitivity and specificity [8]. Sensitivity of an AMS algorithms refers to its ability to detect AT, avoiding a false negative, whereas specificity refers to the absence of AMS during sinus rhythm, avoiding a false positive response. Obviously the greater the sensitivity the lower the specificity. In the clinical setting, arrhythmia related and sensing related issues affect significantly the accuracy of AMS algorithms. Atrial undersensing is responsible for a reduced sensitivity of ATA detection. Two different forms of atrial undersensing have to be distinguished [8]. True atrial undersensing due to insufficient atrial signal amplitude, and functional atrial undersensing due to the coincidence of an atrial signal of sufficient amplitude with an atrial blanking time. The latter is predominantly encountered in atrial flutter. A high programmed atrial sensitivity may cause atrial sensing of far-field signals or noise, whereas a low atrial sensitivity can lead to undersensing during AF. Optimal programming of atrial sensitivity for AMS requires three times the safety margin compared to two times for sinus P waves sensing [10]. Oversensing of ventricular far-field signals (tail end of the QRS complex) represents the most common cause of false positive mode switching (95%), whereas this was rarely (5%) caused by miopotentials. In general, unipolar atrial sensing, paced QRS rhythms, longer dipole lengths, septal and low right atrial implants may predispose to far-field R wave sensing. Any circumstance that prolongs the QRS complex (e.g., flecainide, amiodarone, hyperkalemia) favours such ventriculatrial cross talk. Less commonly, oversensing of atrial signals can occur within the atrioventricular interval [8].

## Automatic mode switching algorithm features

There are three main methods for a device to recognize an atrial arrhythmia [7]:

a) Widely used is a “rate cut-off” criterion: sensed atrial rate exceeding a programmable value (for a defined period of time or cycles) will result in AMS. In the set of these “cut-off” based algorithms atrial rate is continuously monitored by increasing/decreasing counters or by consecutive rapid atrial events counters and, depending on the length of the current atrial interval, AMS is activated when atrial rate exceeds the programmed cut-off criterion. Another variety of this category is the ATA response implemented on the Insignia PM (Guidant Inc., St. Paul, MN, USA). Atrial events above the ATA detection rate increment the detection counter, whereas events below the ATA detection rate decrement the counter. Atrial tachyarrhythmia is detected when the counter reaches a fixed value. Following this, AMS occurs over a programmable time between 1 and 5 minutes, and ventricular rate falls back from the atrioventricular Wenckebach rate to the lower rate or the sensor driven rate. When ATA terminates, the ATA detection counter will decrement with each atrial event below the ATA detection rate. Resynchronization to sinus rhythm occurs when the counter drops from 8 to 0. This device is relatively slow to activate AMS following the onset of ATA, but allows rate smoothing to be effected so as to minimize the beat-to-beat change in pacing rate prior to AMS, and on AMS termination before resynchronization to sinus tracking.

b)Similar high value have the algorithm using a “running average” rate. Such method uses a “mean atrial rate” based on a moving value related to the duration of the prevailing sensed atrial cycle as a criterion to move towards AMS. AMS will occur when the “filtered” or “matched” atrial interval shortens to a predetermined duration. A cut-off or critical value is anyhow defined for the fulfilment of the detection criterion, but it doesn’t refer to the actual atrial interval. This algorithm is used in the Medtronic Thera DR, Kappa 400 and Gem DR implantable defibrillator and the St. Jude Medical families Affinity and Identity. Because the process is gradual, the rapidity of AMS will depend not only on the atrial tachycardia detection rate or interval but also on the pre-existing sinus rate. It is easier for the matched atrial interval to reach the tachycardia detection interval when atrial tachycardia occurs in the setting of a higher resting sinus rate than from a sinus bradycardia. This is because the matching atrial interval starts from a shorter baseline duration on its gradual way to reach the tachycardia detection interval.

In both methods a beat per beat analysis of the atrial coupling is performed for an early detection of the arrhythmia. Some systems are indeed designed to avoid mode switch during atrial ectopic beats or short runs of atrial tachycardia. For example, the Identity (St. Jude Medical, Sylmar, California, USA) pacemaker family uses a “running average” algorithm to define the ATA detection. This filtered rate-based algorithm ensures that AMS event only happens during sustained (although it could be a short one) supra ventricular tachycardia and that the device does not change mode on a single premature atrial contraction.

c) A last method does exist, and shows how sensors can also be used to determine the “physiological” rate of the patient and discriminate with an arrhythmic high rate, [e.g., Diamond/ Clarity (Vitatron BV, Dieren, the Netherlands), SmarTracking of Marathon, (Intermedics, Inc.) and Neway DR (Sorin Biomedica, Saluggia, Italy)]; taking into account the fluctuation in sinus rate, a physiological heart rate range based on the sensor indicated rate is used to define sinus rhythm, and rates beyond the upper range will activate AMS.

For example, the Vitatron Diamond II and Clarity DR AMS is based on a beat-to-beat and sudden onset criterion, independent of a cut-off rate. It detects atrial arrhythmias based on a sudden onset criterion, rather than a critical cut-off rate. Whenever an atrial rate above 100 beats/min and in excess of 15 beats/min with respect to the baseline atrial rate is detected, the AMS is activated. When atrial rhythm shows a sudden increase of at least 15 beats over the current atrial rhythm, the arrhythmia is detected and the pacemaker immediately switches from DDDR to DDIR mode, thus avoiding ventricular tracking of the high abnormal atrial rate.

In recent years complex algorithms have been introduced that may combine two or more methods to fine tune the AMS response and to avoid rapid fluctuation in pacing rate. These combinations of algorithms use additional criteria to distinguish between different types of atrial arrhythmia and other rhythms and are mostly a prerogative of implantable cardioverter defibrillators or dedicated PMs. For example, a P to R relationship and a rate criterion are implemented in the Medtronic AT500 to detect AF and atrial tachycardia.

Another typical example is represented by the Phylos II DR Biotronik Inc. (Berlin, Germany) PM that uses a “retriggerable” atrial refractory period algorithm to provide protection against ATAs. This simple and rapid algorithm continuously extends the total atrial refractory period (TARP) if the PM detects a P wave in the actual TARP (but outside the atrial blanking period), and the atrioventricular interval is not initiated. This causes a DVIR functioning with ventricular based timing. Instantaneous resynchronization occurs when an atrial event occurs outside the TARP, or when the base lower rate is reached. This algorithm provides a sensitive and fast reacting response to onset and termination of ATA, however, it has a low specificity and may result in frequent switching pacing during noise and atrial ectopics. In addition, competitive (asynchronous) atrial pacing occurs during tachycardia, and may paradoxically reinduce AF if AF terminates spontaneously. In addition to this algorithm, a statistical “x of y” criterion is used for detection of ATA. It is detected if a predefined number of atrial sensed intervals x-out-of-y (where y is the length of the “moving” observation window) are shorter than the ATA detection interval. Resynchronization to sinus rhythm occurs when eight consecutive atrial sensing events are below the ATA detection rate or are paced. This “x of y” criteria provides a higher specificity compared to the first retriggerable refractory period method.

Ela Medical (Montrouge, France) also introduced a particularly complex method of “fallback” algorithm, based on a WARAD (Window of Atrial Rate Acceleration Detection), able to prevent high frequency pacing in the ventricle, providing immediate response to pathological rhythm, even in case of partial undersensing, with good sensitivity and specificity. It is based on a double criterion: combines an initial upper rate switch at the onset of ATAs, followed by AMS based on the detection of a sustained atrial rate above a preset ATA detection rate. The WARAD varies and is calculated as a percentage of the preceding PP interval. At the onset, only events outside the window will be sensed and ventricular pacing triggered. This results in a “temporary mode switch”  When 28 of 32 or 36 of 64 consecutive beats above the atrial tachyarrhythmia detection rate are detected, AMS will be initiated (this is called “permanent mode switch”). A further refinement during AMS allows the pacer to function in the DDIR mode for spontaneous ventricular rate less than 100 beats/min, and VVIR mode when this rate is greater than 100 beats/min to avoid atrial competitive pacing. Resynchronization to sinus rhythm occurs if 24 consecutive atrial cycles are less than 110 beats/min. This counter will be reset if premature beats are sensed within this confirmation period until the 24 atrial/ventricular cycles less than 110 beats/min are satisfied.

Some dual chamber pacemakers offer two different AMS algorithms, one for detection of ATA including AF and a supplemental algorithm for the detection of atrial flutter unrecognized by the primary algorithm.

Patients with paroxysmal atrial flutter represent a challenge for AMS algorithms. In several of these patients, a unique form of AMS failure (termed the '2:1 lock-in' phenomenon) can be observed [11]. AMS failure may occur during atrial flutter when alternate flutter waves coincide with the post-ventricular atrial blanking period (locked-in phenomenon). There are little data about the incidence of atrial flutter underdetection by implanted devices with AMS since pacemaker memory functions do not store undetected atrial flutter episodes in contrast to inappropriate AMS. In addition to the mode switching function (atrial tachy response), the Pulsar Max System (Guidant Inc., St. Paul, MN, USA) offers an “atrial flutter response” (AFR). This algorithm starts another refractory period (“AFR window”) of 260 ms (equivalent to an atrial rate of 230 beats/min) if atrial events are sensed within the PVARP. As long as an atrial rate > 230 beats/min is sensed, successive AFR windows start and ventricular pacing is performed independently of atrial sensing, which effectively constitutes AMS to the VDIR mode. Therefore, the AFR provides instantaneous AMS. However, if every second atrial flutter potential occurs during the blanked portion of the PVARP, the AFR will not be able to detect the ATA and 2:1 tracking will persist. No data on the sensitivity and specificity of this ATA detection algorithm has yet been published.

Selection of the suitable algorithm must be done on a case-by-cases basis, considering the following advices based on comparisons and bench testings. Evaluation of AMS performance has to consider the rate of failure of ATA detection (sensitivity) and of inappropriate AMS (specificity), delay between ATA onset and AMS (AMS onset delay), time of pacing at the upper tracking limit, rate during AMS, delay between sinus conversion and return to tracking mode (AMS termination delay), and the number of switches back and forth during one episode (mode oscillations) [7]. In general, a rate cut-off detection method of ATAs provides a rapid AMS onset and resynchronization to sinus rhythm at the termination of ATAs, but may cause intermittent oscillations between the atrial tracking and AMS mode. This can be minimized with a counter of total number of high rate events before the AMS occurs. The use of a running average algorithm results in more stable rate control during AMS by reducing the incidence of oscillations, but at the expense of delayed AMS onset and resynchronization to sinus rhythm [2].

## Clinical implication

**Figure 2:**
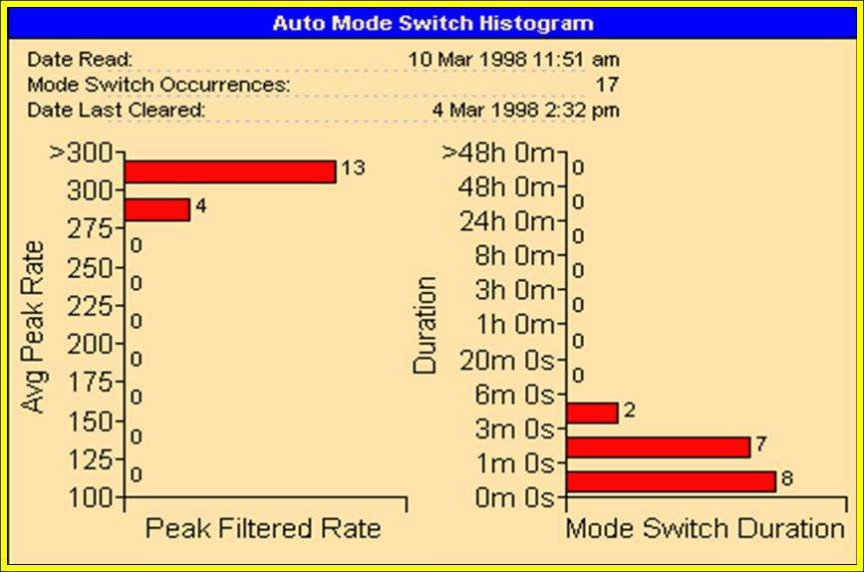
A detail of automatic mode switching histogram of pacemaker St Jude Identity DR, which give information on AMS number, duration and sensed atrial rate during the episodes

**Figure 3:**
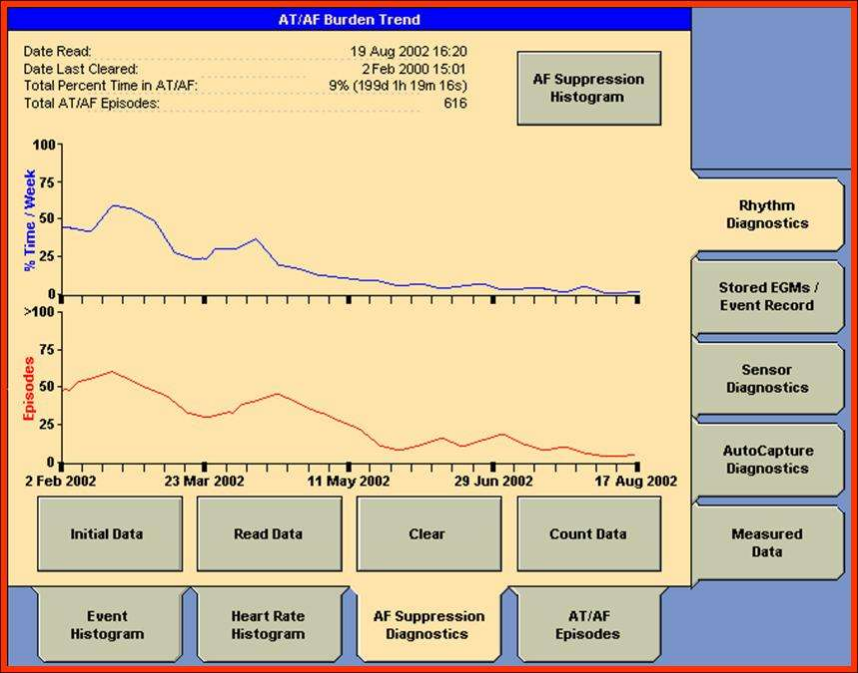
A detail of automatic mode switching histogram of pacemaker St Jude Identity DR, which give information on atrial tachycardia/atrial fibrillation burden trend

Whereas clinical events  (death, stroke, haemorrhage, new or worsening heart failure, etc.) are the type of endpoint of real interest in studies of management of AF, they have been seldom used. The primary reason for not using these endpoints is that they occur with a very low frequency, therefore very large numbers of subjects must be studied in order to show a significant therapy-induced change. This last problem has led to the use of surrogate endpoints in studies to test therapies for AF [12]. The most common used surrogate endpoint to measure rhythm control is the time to first symptomatic recurrence of AF. Automatic mode switching algorithms, which provide data on the time of onset and duration of AMS episodes (Figure 2), allow a more accurate determination of the proportion of time a patient with AF is in AF and have led to the concept of “AF burden” (Figure 3). Moreover, a substudy of a randomized controlled trial of atrial- versus ventricular-based pacing demonstrated that ATA episodes, detected by these algorithms, were associated with increased rates of death and nonfatal stroke [13]. In view of the facts that AMS events are common, have clinical impact, and may be used to verify a therapeutic strategy to control AF, of primary importance is the accuracy of these algorithm in the detection of ATAs. Studies without intracardiac electrograms (EGM) data in PMs patients reported an incidence of ATAs in about 50% of cases using PMs memory data [3,4]. Using conventional diagnostic methods, such as ECG and 24-h Holter monitoring, the incidence of ATAs decreased to 4%-20% in PMs patients [14][15]

Without concomitant ECG or EGM, the sensitivity and the specificity of AMS-related data (event counter, histograms, burden) was unclear. In 40 patients with tachycardia-bradycardia syndrome and Medtronic Thera or Kappa 700 PMs underwent Holter monitoring [16]. Comparison of Holter data with PM interrogation demonstrated that 53 (98.1%) of 54 ATA episodes resulted in AMS. The sensitivity and specificity of AMS for the duration of ATAs were 98.1% and 100%, respectively. A substudy [13] of the Mode Selection Trial (MOST) compared ambulatory monitoring with different PMs atrial high rate episodes data in 47 patients. Five patients had ATA seen on both PMs and ambulatory monitoring, 41 had no arrhythmias with either recording, and one patient had a false positive atrial high rate episode: this gave a sensitivity of 100% and a specificity of 97.6%

**Figure 4:**
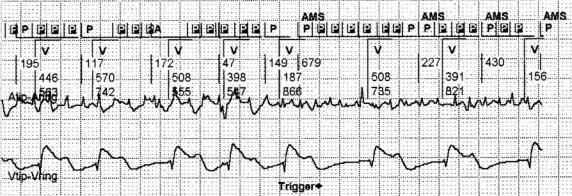
Intracardiac electrograms stored at onset of automatic mode switching

Recently, stored EGMs corresponding to episodes of ATAs have been introduced, thus clarifying the accuracy of AMS in detecting ATAs (Fig. 4). Pollak et al [17] analyzed ATAs episodes in 56 patients with Medtronic Prodigy and Thera model in which some ATAs episodes had a stored atrial EGM snapshot of the atrial tachyarrhythmias. EGM confirmation of ATA correlated with increasing duration and rate of episodes. While only 18% of 44 episodes < 10 seconds in duration and 18% of 56 detected ATA episodes at rate < 250/min were confirmed to be true ATA, atrial high rate episodes at rates > 250/min and > 5 min in duration were confirmed to be true ATA by the stored EGM in 15 (88%) of 17 episodes, although specificity decreased when the programmed atrial sensitivity was increased. Another parameter which affects the specificity of AMS in detecting a true ATA episode is the contiguity, that is the probability of occurrence of another AMS within 5 minutes before or after an AMS. In a study on 24 patients with sick sinus syndrome and paroxysmal AF, implanted with St Jude Identity DR PMs 250 AMS episodes were collected [18]. Intracardiac electrogram recordings were available in each episode to distinguish true arrhythmias from unnecessary AMS. Using the composite criterion of contiguity and length (> 1 min), compared with only the criteria of length, the specificity of AMS was increased from 77.2% to 93.2%, at the cost of 11.9% loss of sensitivity.

Implantable devices as PMs or implantable cardioverter-defibrillators with atrial electrograms provide continuous rhythm monitoring and may thus enhance the diagnostic accuracy of detection of asymptomatic AF [19]. This has been suggested in the Automatic Interpretation for Diagnostic Assistance (AIDA) trial, in which paroxysm of AF lasting more than one minute were recorded by the devices in half of the patients, 58% of these were completely asymptomatic [3]. Recently, in a prospective long-term follow-up study [20] continuous EGM monitoring by an implantable device (Medtronic AT 500) was used for up to 42 months document recurrences of AF and the exact duration of arrhythmia-free intervals and AF episode duration in 110 patients with a class I indication for physiologic pacing and a history of AF. The study demonstrated that asymptomatic AF escaped documentation by ECG recording during follow-up in 59% of patients, moreover 38% of AF recurrences lasting more than 48 h were completely asymptomatic. The underestimated prevalence of recurrent AF, particularly asymptomatic, has obvious clinical implications, the most of which is related to the need of anticoagulation in patients with AF.

Another study [21] evaluated the clinical benefits of PM (Selection, Vitatron) diagnostic function (AF 1.0) in the management of 40 patients with AF and conventional pacing indications. AF recurrences were recordered in 71% of the follow-ups with symptoms reported by patients in only 16%. Thirty-nine percent of therapeutic changes based on conventional assessment were confirmed by AF 1.0 data, and in 61% of instances, the initial changes were modified by AF 1.0. The authors concluded that PM diagnostic functions offered a unique documentation of AF in asymptomatic patients, and allowed therapeutic adjustments impossible otherwise.

Finally on clinical basis, the symptomatic benefit of AMS is related to improvement of tachycardia related symptoms by avoiding a rapidly paced ventricular rate at the onset of an atrial tachycardia. A randomized, crossover, prospective study [1] compared three pacing modalities-DDDR with mode switching (DM), DDDR with conventional upper rate behaviour (DR) and VVIR (VR)-in patients with a history of ATAs, and to assessed the efficacy of six AMS algorithms. Forty-eight patients with a history of ATAs and heart block had a DM pacemaker implanted. Pacemakers were programmed to DM, DR and VR modes for 4 weeks each in a randomized crossover design. All subjects used a patient-activated electrocardiographic (ECG) recorder throughout the study and additionally underwent ambulatory ECG monitoring and a treadmill exercise test in each mode. They completed three symptom questionnaires at the end of each pacing period. At the end of the study, patients chose their preferred pacing period. DM was significantly better than VR mode objectively (exercise time DM 8.1 min, VR 7.0 min, p < 0.01) and subjectively (perceived well-being DM 69, VR 51, p < 0.001; functional class DM 2.2, VR 2.5, p < 0.05; subjective symptom score DM 21.2, VR 26.8, p = 0.01). Patient-perceived well-being was significantly better with DM than with DR mode (DM 69, DR 60, p = 0.02). DM mode was the preferred pacing period (DM 51%, DR 14%, VR 14%). Early termination of pacing because of adverse symptoms was requested by 33% of patients during VR, 19% during DR but only 3% during DM mode. A higher proportion of patients with a fast mode-switching device preferred DM mode (fast 55%, slow 49%), whereas no patients with a fast mode-switching device chose VR as the preferred mode (fast 0%, slow 19%). In the subgroup of patients who had had atrioventricular node ablation, DM was also preferred to VR mode (DM 53%, VR 27%). Overall, there were only two cases of inappropriate mode switching and one case of inappropriate tracking of an ATA. Significantly, the authors concluded that DDDR PM with mode switching is the pacing mode of choice of patients with paroxysmal ATAs.

## Figures and Tables

**Figure 1:**
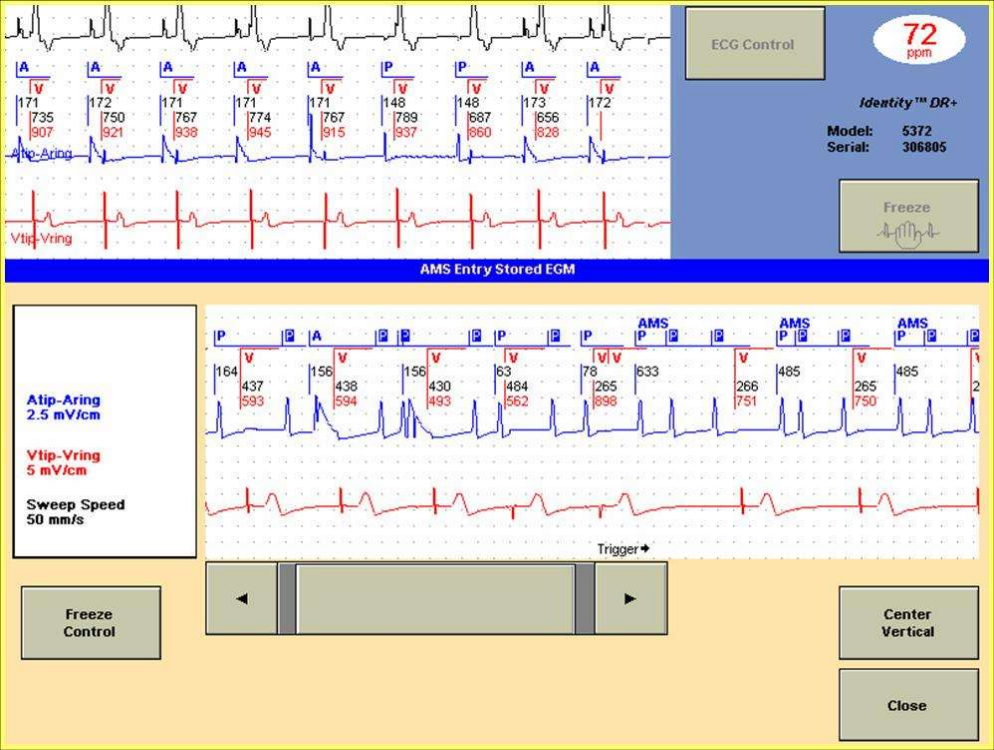
In a patient with a paroxysmal atrial fibrillation the automatic mode switching (AMS) entry stored intracardiac electrogram showed the activation of AMS algorithm

**Table 1:** Parameters which influence an appropriate mode switching

Characteristics	Remarks
Characteristics of arrhythmia	Atrial rate, coexisting atrial flutter, atrial amplitude electrogram
Programmed parameters	Atrial sensing, atrial post-ventricular blanking period
Characteristics of the AMS algorithm	Accuracy, Onset, AMS response, Resynchronization

## References

[1] Kamalvand K, Tan K, Kotsakis A (1997). Is mode switching beneficial: A randomized study in patients with atrial tachyarrhythmias. J Am Coll Cardiol.

[2] Lau CK, Leung SK, Tse HF (2002). Automatic mode switching of implantable pacemakers: II. Clinical performance of current algorithms and their programming. Pacing Clin Electrophysiol.

[3] Garrigue S, Cazeau S, Ritter P (1996). Incidence of atrial arrhythmia in patients with long term dual-chamber pacemakers. Arch Mal Coeur Vaiss.

[4] Defaye P, Dournaux F, Mounton E (1998). Prevalence of supraventricular arrhythmias from the automatic analysis of data stored in the DDD pacemakers of 617 patients: The AIDA study. Pacing Clin Electrophysiol.

[5] Swerdlow CD, Schsls W, Dijkman B (2000). Detection of atrial fibrillation and flutter by a dual-chamber implantable cardioverter-defibrillator. For the Worldwide Jewel AF Investigators. Circulation.

[6] Ellenbogen KA, Mond HG, Wood MA (1997). Failure of automatic mode switching: recognition and management. Pacing Clin Electrophysiol.

[7] Israel CW (2002). Analysis of mode switching algorithms in dual chamber pacemakers. Pacing Clin Electrophysiol.

[8] Lau CK, Leung SK, Tse HF (2002). Automatic mode switching of implantable pacemakers: I. Principles of instrumentation, clinical, and hemodynamic consideration. Pacing Clin Electrophysiol.

[9] Brunnel-Larocca HP, Rickli H, Weilenmann D (2000). Importance of ventricular rate after mode switching during low intensity exercise as assessed by clinical symptoms and ventilatory gas exchange. Pacing Clin Electrophysiol.

[10] Leung SK, Lau CP, Lam CT (1998). Programmed atrial sensitivity: A critical determinant in atrial fibrillation detection and optimal automatic mode switching. Pacing Clin Electrophysiol.

[11] Goethals M, Timmermans W, Geelen P (2003). Mode switching failure during atrial flutter: the “2:1 lock-in” phenomenon. Europace.

[12] Wyse DG (2002). Selection of endpoints in atrial fibrillation studies. J Cardiovasc Electrophysiol.

[13] Glotzer T, Hellkamp A, Zimmerman J (2003). Atrial high rate episodes detected by pacemaker diagnostics predict death and stroke. Report of the Atrial Diagnostics Ancillary Study of the Mode Selection Trial (MOST). Circulation.

[14] Detollenaere M, van Wassenhove E, Jordaens L (1992). Atrial arrhythmias in dual chamber pacing and their influence on long-term mortality. Pacing Clin Electrophysiol.

[15] Chamberlain-Webber R, Petersen MEV, Bries AI (1994). Reason for reprogramming dual chamber pacemakers to VVI mode: a retrospective review using a computer database. Pacing Clin Electrophysiol.

[16] Passman RS, Weinberg KM, Freher M (2004). Accuracy of mode switch algorithms for detection of atrial tachyarrhythmias. J Cardiovasc Electrophysiol.

[17] Pollak WM, Simmons JD, Interian A (2001). Clinical utility of intraatrial pacemaker stored electrograms to diagnose atrial fibrillation and flutter. Pacing Clin Electrophysiol.

[18] De Simone A, Senatore G, Turco P (2005). Specificity of atrial mode switching in detecting atrial fibrillation episodes: Roles of length and continuity. Pacing Clin Electrophysiol.

[19] Isreael CW, Barold SS (2001). Pacemakers systems as implantable cardiac rhythm monitors. Am J Cardiol.

[20] Isreael CW, Gronefeld G, Ehrlich J (2004). Long-term risk of recurrent atrial fibrillation as documented by an implantable monitoring device. J Am Coll Cardiol.

[21] Fauchier L, Briand F, Soto FX (2003). Management of atrial tachyarrhythmias: Benefits of pacemakers diagnostics. Pacing Clin Electrophysiol.

